# The DNA Topoisomerase 1 Contributes to Stress Response in *Saccharomyces cerevisiae*, Regardless Its Catalytic Activity

**DOI:** 10.3390/biology14050499

**Published:** 2025-05-03

**Authors:** Anna D’Alfonso, Alessandra Egidi, Ludovica Proietti, Giorgio Camilloni

**Affiliations:** Dipartimento di Biologia e Biotecnologie, Università degli studi di Roma, Sapienza, 00185 Rome, Italy; anna.dalfonso@uniroma1.it (A.D.); ale.egidi.89@gmail.com (A.E.); ludovica.proietti95@gmail.com (L.P.)

**Keywords:** *Saccharomyces cerevisiae*, stress genes, DNA topoisomerase 1, Rpd3, G-quadruplex

## Abstract

In this study, we examined the activation of the ATG8, HSP12, KGD1, and POT1 genes in response to decreased glucose levels in the culture medium. Our results show that in *top1Δ* strains, gene activation is further enhanced compared to WT strains under low glucose conditions, indicating that Top1p represses these genes. This repression occurs independently of its catalytic function. We investigated Rpd3p as an interacting factor of Top1p and found that in *rpd3Δ* mutants, gene expression under low glucose conditions is even higher than in *top1Δ* strains, suggesting that Rpd3p also acts as a negative regulator. ChIP analysis revealed that while Top1p levels in regulatory regions remain constant, Rpd3 recruitment increases on promoters after glucose reduction in WT strains but significantly decreases in *top1Δ* strains. Overall, our findings suggest that Rpd3p is recruited by Top1p to regulate gene expression at controlled physiological levels, highlighting the role of Top1p in transcriptional regulation, controlling helical stress, and interacting with key regulatory factors in response to environmental changes.

## Simple Summary

In this work, we observed how DNA topoisomerase 1 can reduce the transcriptional response of certain stress genes. DNA topoisomerase 1, due to its catalytic activity, can relax supercoiled DNA, but in these observations, it seems that this catalysis is not required; rather, its scaffold activity is necessary to recruit other regulatory factors to the promoter. In this regard, we observed the involvement of Rpd3p, which usually acts as a transcriptional repressor, and we hypothesized that DNA topoisomerase 1 recruits this factor to the transcriptional machinery. The role of possible alternative structures in recruiting DNA topoisomerase 1 is also hypothesized. This work highlights DNA topoisomerase 1 ability to function both as an enzyme that relaxes DNA and as a scaffold protein.

## 1. Introduction

Basic DNA transactions such as replication, transcription and recombination, require the double helix opening. When the DNA strands come apart, in a closed topological domain, superhelical stress is generated. This latter process can affect various structural and functional DNA features, such as the formation of alternative structures (cruciform, slippage, Z-DNA, G-quadruplex) or cause mechanical distortions, leading to replication fork arrest and/or hindering of transcription elongation [1]. To solve the topological problems due to DNA transactions, specific enzymes have evolved: DNA topoisomerases [2]. These proteins, by nicking and closing the DNA strands, allow the release of topological stress. Depending on whether a single- or double-stranded cut is produced and subsequently re-joined, the DNA topoisomerases are accordingly divided into type I or II. Therefore, DNA topoisomerases are recognized primarily for their actions on removing the torsional stress of DNA molecules that undergo basic metabolism [3]. Previous experiments, however, indicate that, in eukaryotes, a particular type of DNA topoisomerase I, defined as “IB” (commonly called Top1p), can regulate different functions beyond its catalytic activity, as reported for transcription initiation by RNA polymerase II [4,5] in mammalian cells, or as transcriptional repressor during stationary phase [6] or rDNA silencing [7,8] in *Saccharomyces cerevisiae*.

When yeast cells are challenged with sudden environmental changes such as heat shock, oxidative stress, glucose starvation or high osmolarity, they respond with an environmental stress response (ESR), a dramatic change of gene expression including high transcription of defined stress genes [9]. In particular, the ADH2 gene, becomes highly transcribed in *top1Δ* mutants relative to WT when glucose concentration in the growth medium is reduced [10]. This repressive role of *TOP1* on *ADH2* expression is reminiscent of a (unknown) mechanism that represses a series of genes during stationary phase expression in *S. cerevisiae* [6]. More recently it has been demonstrated that ncRNAs transcription by RNA polymerase II at ITS regions of rDNA, is silenced by the combined activity of both DNA topoisomerase I and Sir2 proteins. Top1p recruits Sir2p to ribosomal genes beside its catalytic activity, chromatin became deacetylated and the repressive structure determines ncRNA silencing [8].

In this work, we analyzed the transcriptional activity of a specific set of stress genes when glucose is reduced in the growth medium, and we compared the transcriptional response in WT and *top1Δ* mutants. We also evaluated the relevance of the DNA topoisomerase 1 catalytic activity in transcriptional control. We conclude that the repressive complex containing Rpd3 histone deacetylase is recruited by Top1p, regardless of its catalytic activity, at the promoter of the studied genes. The repressive, low-acetylated state of chromatin, as revealed by Chromatin IP experiments, is maintained. These observations strongly support the scaffold role of DNA topoisomerase IB in the recruitment of transcriptional regulators of the RNA polymerase II machinery.

## 2. Materials and Methods

### 2.1. Yeast Strains and Growth Conditions

The strains used in this work are WT (W303: MAT a, ade2-1, ura3-1, his3-11,15, trp1-1, leu2-3, 112, can1-100); *top1∆* (W303; top1::LEU2); TOP1-HA (W303; TOP1 + 3HA; HIS5+); *rpd3∆* (W303; rpd3::LEU2).

We used two different grow media: “standard glucose” medium (1% Yeast Nitrogen Base, 2% glucose, 1% amino acids according to each strain’s auxotrophy) and “low glucose” medium (1% Yeast Nitrogen Base, 0.05% glucose [11], 1% amino acids according to each strain’s auxotrophy).

Cultures were grown according to standard protocols [12] in “standard glucose” medium until exponential phase.

Then, a volume of culture was collected and used as the “standard glucose” sample (2% glucose in the medium-2% glu sample).

The same volume of cells was collected from the original culture, washed to remove the medium, and resuspended in the identical volume of “low glucose” medium (containing 0.05% glucose as explained above). These cells were incubated at 28 °C for 3 h and at the end of the treatment, were collected to represent the “low glucose” sample (0.05% glu sample).

Plasmids used to transform the *top1∆* strain are YCp50 (URA+), empty vector, YCp50+ yTOP1 (URA+) and YCp50+ ytop1Y727F (URA+) which constitutively express yeast TOP1 or top1Y727F noncatalytic form respectively [13].

### 2.2. Gene Expression

RNA from 3 × 10^8^ cells was obtained according to standard protocols [10]: 1 μg of total RNA was subjected to DNase I treatment and then to reverse transcription using iScriptTM cDNA synthesis kit, Bio-Rad Laboratories, Inc. 2000 Alfred Nobel Drive, Hercules, CA 94547, USA.

An amount of the resulting cDNA (corresponding to 50 ng of original RNA) was amplified with the Bio-rad Mini Opticon Real-time PCR system, using Bio-rad Sso Advanced SYBR Green Supermix and specific primer pairs (see primers section) to determine Ct of analyzed cDNAs. The ∆Ct values were calculated respect to the housekeeping gene ACT1.

### 2.3. Chromatin Immunoprecipitation

Cells were harvested as reported in the growth condition section.

3 × 10^9^ cells were crosslinked with 1% formaldehyde at room temperature (30 min incubation time for Top1-HA IP, 45 min for Rpd3 IP, 15 min for pan-acH3). 330 mM Glycine was added for 5 min to quench formaldehyde. Cells were then submitted to the Chromatin Immuno-Precipitation (ChIP) procedure according to the protocols described [14]. Briefly, cells were collected, the medium removed, and then they were washed with PBS (140 mM NaCl, 2.5 mM KCl, 8.1 mM Na_2_HPO_4_). Cells were then lysed under vigorous shaking with HCl-treated glass beads for 1 h in the presence of PBS supplemented with 1.5 mM NaCl, 1 mM EDTA, 1% Triton X-100, 0.1% SDS, and protease inhibitors at 4 °C.

Extracts were then subjected to ultrasonic shearing to randomly fragment the DNA. Sonication was performed at the amplitude needed to obtain an average of fragments nearly sized to 500 bp. Proteins were quantified using Bradford assay.

300 ng of proteins were collected to be used as the INPUT, not immunoprecipitated sample; the same amount of sample was used as the IP, immunoprecipitated sample.

IPs were incubated with 3 μg antibodies (SantaCruz Anti-HA, Abcam Anti-Rpd3 or Abcam Anti-pan-acH3) in presence of 140 mM NaCl, 100 μg of Bovine Serum Albumine, at 4 °C overnight. Chromatin antibody complexes were titrated incubating samples with protein A Sepharose beads (Amersham, GE Healthcare, Chiltern, UK) for 2 h at 4 °C.

Samples were then subjected to sequential washing with different buffers:

(1) 140 mM NaCl, 2.5 mM KCl, 8.1 mM Na_2_HPO_4_, 1.5 mM NaCl, 1 mM EDTA, 1% TritonX-100, 0.1% SDS;

(2) 275 mM NaCl, 2.5 mM KCl, 8.1 mM Na_2_HPO_4_, 1.5 mM NaCl, 1 mM EDTA, 1% TritonX-100, 0.1% SDS;

(3) 500 mM NaCl, 2.5 mM KCl, 8.1 mM Na_2_HPO_4_, 1.5 mM NaCl, 1 mM EDTA, 1% TritonX-100, 0.1% SDS;

(4) 10 mM Tris HCl pH 8; 250 mm LiCl; 0.5% NP-40; 0.5% Na dehoxycholate;

(5) 10 mM Tris- HCl pH 8; 1 mM EDTA.

A final 15-min-long treatment with 50 mM Tris-HCl pH 8, 10 mM EDTA, 1% SDS at 65 °C of temperature permitted to remove the DNA/protein complexes from the binding with antibody and protein A- Sepharose beads.

Cross bindings inserted by formaldehyde were removed incubating samples (IPs and INPUTs) at 65 °C for at least 6 h.

Ip samples and INPUT samples were subjected at this point to the standard procedures of DNA purification.

The obtained DNAs were resuspended in ddH_2_O.

Amplification was performed with the Bio-rad Mini Opticon Real-Time PCR system using the specific primer pairs for promoter analyses (see the primers section) to obtain the Ct of the analysed regions. The ∆∆Ct values of IP samples were calculated respect to rDNA ARS signal [8], rDNA 37S promoter [15], ARS 504 [16], for acH3, Top1 and Rpd3 IPs respectively, and to the corresponding INPUT signal.

### 2.4. Primers:

#### 2.4.1. Gene Expression Analysis

ATG8F: 5′ GTGATTTGCGAAAAAGCTG

ATG8R: 5′ CTTCTCAGGGGGTAGCATA

HSP12F: 5′ AGTCATACGCTGAACAAGGT

HSP12R: 5′ CGTTATCCTTGCCTTTTTCG

KGD1F: 5′ GCCAGAAGGTTTTGAAGTGC

KGD1R: 5′ CATCTTCACCGGAAACCCTA

POT1F: 5′ GGTCCGTAGCCAACCAGTTA

POT1R: 5′ CCGCGAATGCTTCATTTATT

ACT1F: 5′ ACGTTCCAGCCTTCTACGTTTCCA

ACT1R: 5′ AGTCAGTCAAATCTCTACCGGCCA

#### 2.4.2. Primers for Chromatin IP Analysis

ATG8prmF: 5′ GTTGAGGAGGGGATTGATAAGAGA

ATG8prmR: 5′ AACCTGTCAGCAATCCTCTCCGAC

HSP12prmF: 5′ ACGTATAAATAGGACGGTGAATTGC

HSP12prmR: 5′ TTCAGAAGCTTTTTCACCGAATC

KGD1prmF: 5′ TGTTTCTGTCACATAGTCGCAGCA

KGD1prmR: 5′ GAAGACACGAACCTTAGCATAACG

POT1prmF: 5′ CAAAGGGAAACGGGGATAATAGTA

POT1prmR: 5′ CTCCACCAAATGATCCTTGATACT

rDNA-ARSF: 5′ TCAGATGAAAGATGAATAGACATAGGA

rDNA-ARSR: 5′ AAAGTAACATCCCAATGCGG

rDNA-37SpromF: 5′ TCAGATGAAAGATGAATAGACATAGGA

rDNA-37SpromR: 5′ AAAGTAACATCCCAATGCGG

ARS-504 F: 5′ GTCAGACCTGTTCCTTTAAGAGG

ARS-504 R: 5′ CATACCCTCGGGTCAAACAC

### 2.5. Statistical Analysis

For both gene expressions and chromatin immunoprecipitations, data reported in figures were calculated in the following way: we calculated fold increases relative to “low glucose” samples or “standard glucose” samples first (see the relative materials and methods section to clarify the reporter gene used for each specific experimental procedure). Then the ratios between “low glucose” and “standard glucose” fold increases were calculated (as a result the amount of “standard glucose” becomes 1). Finally, we graphed the means of at least three biological replicates, with the relative standard deviations. *p*-values were calculated using Student’s *t*-test (* *p* ≤ 0.05; ** *p* ≤ 0.01).

## 3. Results

To highlight the molecular mechanisms that cells utilize to induce a stress response, we decided to study the behaviour of specific stress genes after shifting cells from “standard glucose” (2%) to “low glucose” (0.05%). For all procedures, cells were treated and collected as described in the Materials and Methods section.

Based on previous studies, we chose to investigate the genes *ATG8*, *HSP12*, *KGD1*, and *POT1*.

*ATG8* is involved in autophagosome formation [17]. *HSP12*, a transmembrane heat shock protein, is known to participate in the cellular response to major stresses, including glucose depletion [18]. *KGD1*, a subunit of alpha-ketoglutarate dehydrogenase, is a key enzyme in the TCA cycle [19]. *POT1* is the enzyme responsible for producing acetyl-CoA via the fatty acid beta-oxidation pathway [20].

### 3.1. Decrease in Glucose Concentration Leads to Changes in the Expression of Analysed Stress Genes

We measured RNA transcription of the selected genes (*ATG8*, *HSP12*, *KGD1*, and *POT1*) in wild-type (WT) cells, comparing conditions with 2% and 0.05% glucose to identify any changes in expression. Yeast WT cultures were grown in “standard glucose” and then in “low glucose” according with methodology explained in materials and methods section. RNA, and subsequently the corresponding cDNA from both samples, was prepared as described in the Materials and Methods section.

The expression levels of *ATG8*, *HSP12*, *KGD1*, and *POT1* were measured by qRT-PCR. The ΔCt values were calculated relative to *ACT1*, and fold increases of the 0.05% glucose samples were normalized to those obtained from the 2% glucose samples.

**Figure 1:**
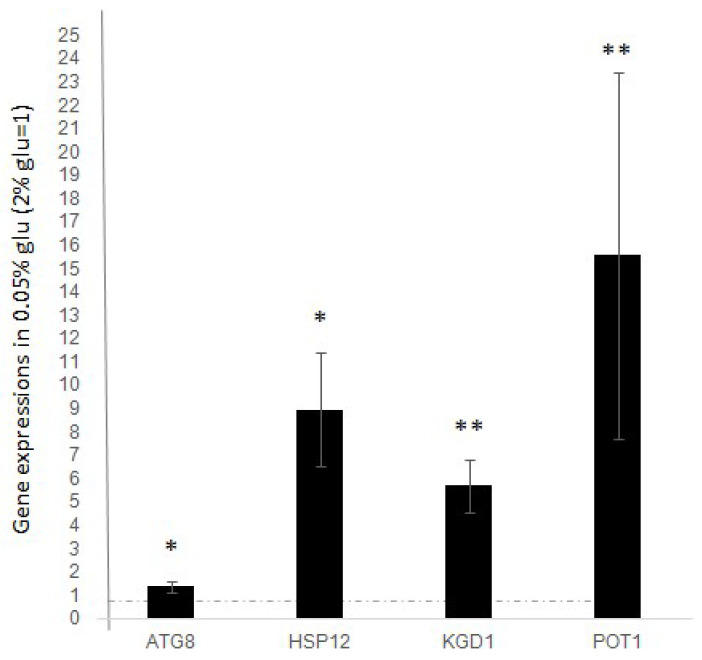
Expression level of ATG8, HSP12, KGD1, POT1 after glucose deprivation (0.05% glucose). Fold increase is calculated respect to ACT1 expression. Dashed line represents expression level before glucose deprivation (2% glucose). The values and statistics were calculated from three biological replicates. Significance was assessed using Student’s *t*-test. * *p* ≤ 0.05, ** *p* ≤ 0.01.

As shown in Figure 1, we observed a significant increase in the expression levels of all analysed genes after the decrease in glucose concentration. Compared to expression levels in standard glucose (dashed line), we found that all the analyzed genes significantly increase their expression after stimulus, indicating that the expression of *ATG8*, *HSP12*, *KGD1*, and *POT1* is positively regulated during the response to stress induced by glucose deprivation.

### 3.2. Glucose Reduction Induces H3 Acetylation in Stress Gene Promoters

Histone acetylation is known to be strongly related to gene expression [21]. In particular, it has been demonstrated that certain stress conditions (such as oxidative exposure or aging) affect the expression levels of corresponding stress response genes through H3 acetylation of their promoter regions [16].

**Figure 2:**
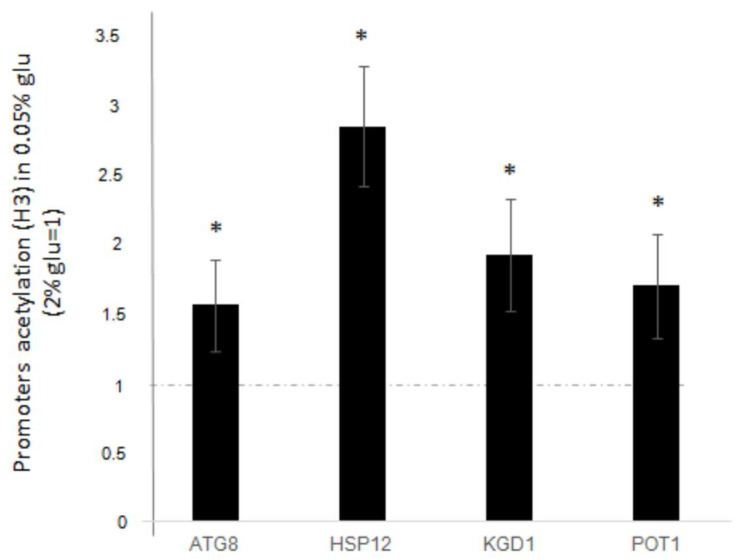
H3 global acetylation at promoter regions of ATG8, HSP12, KGD1 and POT1 genes, after glucose deprivation (0.05% glucose). Fold increases are calculated respect to rDNA-ARS region and relative INPUT. Dashed line represents H3 acetylation before glucose deprivation (2%glucose). The values and statistics were calculated from three biological replicates. Significance was assessed using Student’s *t*-test. * *p* ≤ 0.05.

We wanted to clarify whether the upregulation of genes, following glucose deprivation, correlates with H3 acetylation in the promoter regions of these specific genes. Thus, we performed a ChIP analysis using antibodies against globally acetylated H3 histone on the promoters of *ATG8*, *HSP12*, *KGD1*, and *POT1*. Samples were treated as described in the Materials and Methods and then subjected to qPCR using appropriate primers (listed in the Materials and Methods). The ∆∆Ct values from qPCR were against rDNA-ARS [8], comparing the IP and INPUT signals. The resulting fold increase values after glucose deprivation (0.05%) were compared to those obtained from standard glucose growth (2%). Data presented in Figure 2 show a significant increase in the amount of H3 acetylation after glucose deprivation compared to standard glucose growth. The increase ranges from approximately 1.5 to 3-fold for all analysed genes.

### 3.3. The Catalytic Activity of Top1p Is Not Required for the Expression Activation

Previous studies have described how *TOP1* depletion affects the expression of genes involved in responses to various stresses, such as aging [6,22]. To investigate whether Top1p influences also the expression of the genes *ATG8*, *HSP12*, *KGD1*, and *POT1* in the context of glucose reduction, we examined strains lacking *TOP1* gene or lacking the catalytically active version of the enzyme. We transformed *top1Δ* cells with a plasmid carrying the wild-type *TOP1* gene cloned in the YCp50 vector (YCp50yTOP1), mimicking the WT condition. The “non-*TOP1* condition” is represented by the *top1Δ* strain transformed with the empty YCp50 vector (YCP50 e.v.). Additionally, we used a *top1Δ* strain transformed with a plasmid that constitutively expresses a mutated form of the TOP1 gene, which produces a protein where the single amino acid Tyrosine in the active site is replaced by Phenylalanine, resulting in a full-length non-catalytic enzyme known as *Y727Ftop1* [23], cloned into YCp50 (YCp50y*top1Y727F* here also defined as “YF”). See the materials and methods section for plasmids.

**Figure 3:**
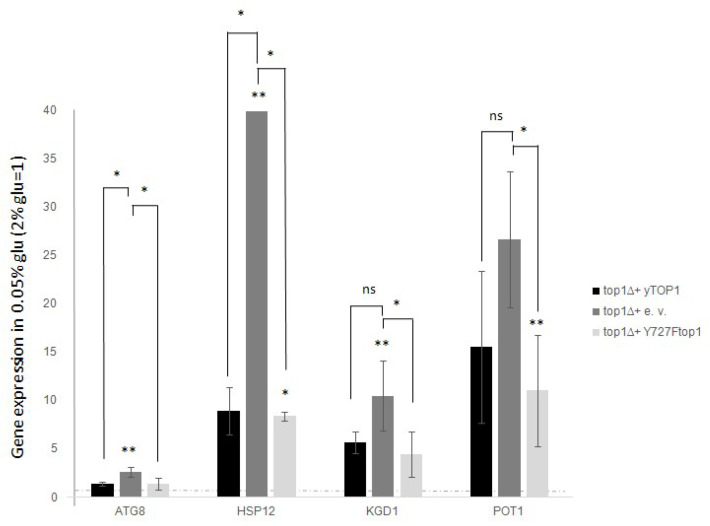
In top1∆ strain the expression intensifies for all the analysed genes after glucose deprivation (0.05% glucose). Conversely, not catalytic top1 mutant restore the WT expression fold increase. top1∆ strain was added with a Ycp50 empty vector, or a Ycp50 + yTOP1 gene, here considered as WT, or Ycp50 + Y727Ftop1 not catalytic form of TOP1 (see materials and methods section). Dashed line represent levels before glucose deprivation (2% glucose). Black bars are WT cells (top1∆ added with Ycp50-yTOP1), grey bars are top1∆ samples (added with Ycp50 empty vector). Light grey bars are YF cells (added with Ycp50 + Y727Ftop1) The values and statistics were calculated from three biological replicates. Significance was assessed using Student’s *t*-test. * *p* ≤ 0.05, ** *p* ≤ 0.01. ns = not significant.

In this context, to determine the relevance of catalytic activity in gene expression following glucose reduction, we assessed the expression of *ATG8*, *HSP12*, *KGD1*, and *POT1* in the “*top1Δ*”, “WT” and “YF” strains. We employed the previously described approach: we grew the cells in standard glucose, then shifted them to low glucose for 3 h, and finally prepared RNA from cells grown under both conditions. The mRNA levels of the selected genes were measured by qRT-PCR with ACT1 as a reference. Fold increases in the 0.05% glucose samples were normalized to those obtained from the 2% glucose samples (Figure 3, WT black histograms). For *ATG8*, and *HSP12*, we observed a significantly enhanced activation in the *top1Δ* samples compared to WT (Figure 3, *top1Δ* dark grey histograms). In contrast, for *KGD1* and *POT1*, we observed slight and non-significant differences between the *top1Δ* and WT strains.

When comparing *top1Δ*, WT, and YF strains regarding their transcription of the studied genes, we found that complementing the *top1* deletion with either WT or YF alleles yielded similar results: gene expression after glucose reduction was lower than in the *top1Δ* strain, suggesting that catalytic activity is not relevant in the observed phenotype (see Figure 3, black bars compared to dark or light grey bars).

It is well established from various studies that the Top1 protein has the unique ability to act independently of its catalytic function, particularly under conditions where it serves as a recruiter of other factors in various chromatin contexts and/or different regions of the genome [4,5,8,24,25,26]. This characteristic behavior is referred to as “Top1 scaffold activity” to distinguish it from its purely enzymatic activity. Thus, these findings reported in Figure 3 indicate that variation of the expression levels depend on the presence or absence of Top1p, not on its enzymatic activity.

### 3.4. After Glucose Deprivation, the Non-Catalytic Top1p Restores H3 Acetylation in the WT Strain, Which Is Significantly Increased by TOP1 Deletion

**Figure 4:**
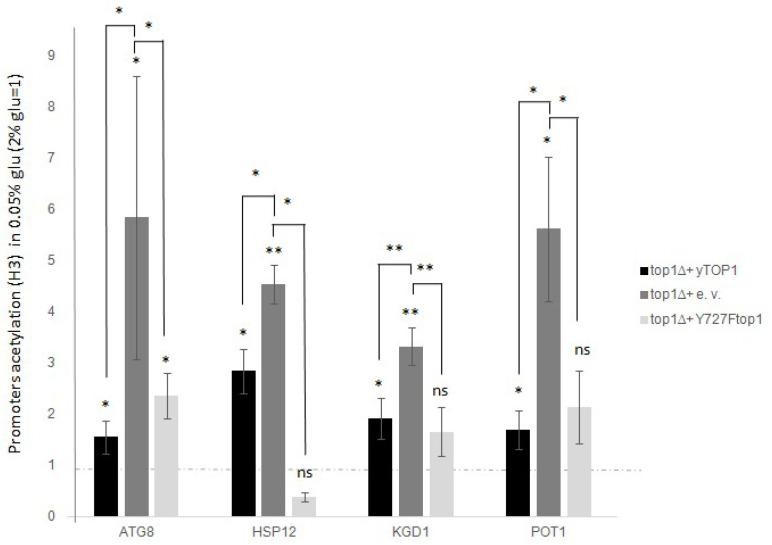
top1∆ intensify H3 global acetylation of all the analysed promoter genes after glucose deprivation (0.05% glucose). Not catalytic top1 mutant restore the WT H3 global acetylation. top1∆ strain was added with a Ycp50 empty vector, or a Ycp50 + yTOP1 gene, here considered as WT, or Ycp50 + Y727Ftop1 not catalytic form of TOP1 (see materials and methods section). Dashed lines represent levels before glucose deprivation (2% glucose). Black bars are WT cells (top1∆ added with Ycp50-yTOP1), dark grey bars are top1∆ samples (added with Ycp50 empty vector), light grey bars are top1∆ cells added with Ycp50 + Y727Ftop1 not catalytic version. The values and statistics were calculated from three biological replicates. Significance was assessed using Student’s *t*-test. * *p* ≤ 0.05, ** *p* ≤ 0.01. ns = not significant.

Considering the results obtained from studying the transcription of stress genes, we aimed to determine whether Top1p could interfere in the regulation of expression following glucose deprivation, also involving changes in H3 acetylation. To assess this, we quantified the global H3 acetylation rate at promoters in the *top1∆* mutant after glucose deprivation, and in the *top1∆* mutant transformed with the *TOP1* WT gene or with the non-catalytic version *Y727Ftop1*, as described above. We then compared these data to those from standard glucose growth and from the WT strain. The analysis was performed as previously described, and the results are shown in Figure 4.

Comparing data from WT cells and *top1∆* mutants, we observed a significantly higher increase in H3 acetylation following glucose deprivation, with an average increase of 2–5-fold for all analyzed genes (Figure 4, dark grey histogram).

The results were also obtained by measuring the amount of H3 acetylation in *top1∆* cells supplemented with *Y727Ftop1* (Figure 4, light grey histogram), which showed that the non-catalytic Top1p version can restore the WT level of acetylation. This occurs at the promoters of all analyzed genes, regardless of Top1p catalytic activity, but solely based on its presence or absence.

Altogether these data, suggest that Top1p also contributes to the H3 acetylation rate independently of its catalytic function.

### 3.5. The Presence of Top1p at Stress Gene Promoters Does Not Change After Low Glucose Treatment in WT Cells

Motivated by the idea that the Top1 protein could act as a negative regulator of gene expression due to its scaffold role, we hypothesized that we would find Top1p on promoters under conditions of repression (standard glucose conditions) but absent when genes are actively transcribed (low glucose conditions).

To test this hypothesis, we conducted a ChIP analysis of Top1p at the promoters of the *ATG8*, *HSP12*, *KGD1*, and *POT1* genes, in growing conditions of standard glucose or after glucose deprivation, and compared the results, accordingly, as described in the Materials and Methods section. The ChIP analysis was performed using a WT strain in which the *TOP1* gene was tagged with 3HA, producing a Top1-3HA fusion protein, which is detectable with anti-HA antibody. After immunoprecipitation and recovery of DNA, samples were subjected to qPCR: ∆Ct values were obtained between IP and INPUT samples and then fold increases of the “low glucose” samples were compared to the “standard glucose” samples. The rDNA 37S promoter (rDNA-37Sprom) was used as a reporter for Top1p immunoprecipitation.

**Figure 5:**
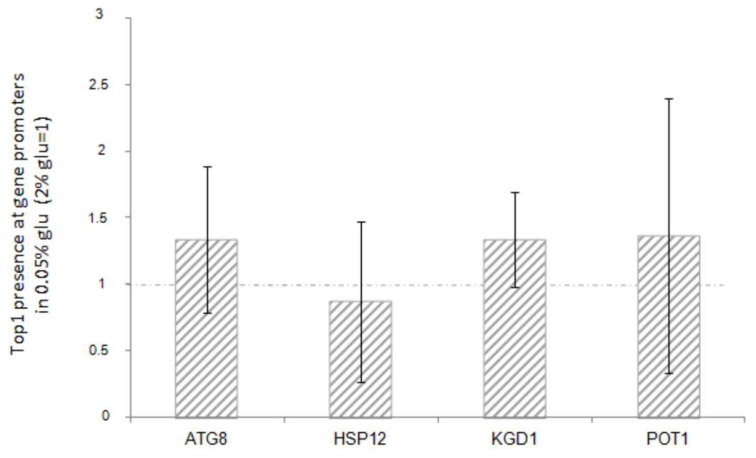
ChIP analysis of Top1 protein at ATG8, HSP12, KGD1, POT1 promoters after glucose deprivation (0.05% glucose). The IP was performed using anti-HA antibody, in a WT strain where the TOP1 gene was 3-HA tagged (see materials and methods). Fold increases are calculated respect to rDNA-37S promoter region and to relative INPUT. Dashed line represents Top1 enrichment before the glucose deprivation (2% glucose). The values and statistics were calculated from three biological replicates. Significance was assessed using Student’s *t*-test.

The results reported in Figure 5 show that there is no significant variation in the amount of Top1 protein bound to the promoters of all the genes analysed in this study, before or after glucose reduction.

Furthermore, there is no correlation with the transcriptional increase observed in each strain. This suggests that the activation or repression of the downstream genes may not be directly associated with the presence of Top1p itself.

### 3.6. The Absence of Rpd3p Affects Expression Changes After Glucose Depletion

An interesting interactor of Top1p is the Rpd3 histone deacetylase [27], which contributes to both the repression and activation of target genes. It acts within two different complexes known as the Large complex and the Small complex. These complexes are responsible for transcription repression and activation, respectively. The distinctive component of the Large complex is the Pho23 factor [28], which is also known to be a Top1p interactor [29].

To determine whether Rpd3p really influences the regulation of the analysed genes by its presence, we measured their expression levels in a *rpd3∆* strain before and after glucose deprivation and compared the results to those observed in the wild-type (WT) strain.

As described in previous sections, cells were grown in a medium with a standard concentration of glucose (2% glucose), then shifted to a medium containing a low glucose concentration (0.05% glucose) for three hours. RNA was extracted and converted into cDNA. Expression levels were measured by qRT-PCR using *ACT1* to calculate the ∆Ct. Fold change values for the 0.05% glucose samples were normalized to those obtained for the 2% glucose corresponding samples.

**Figure 6:**
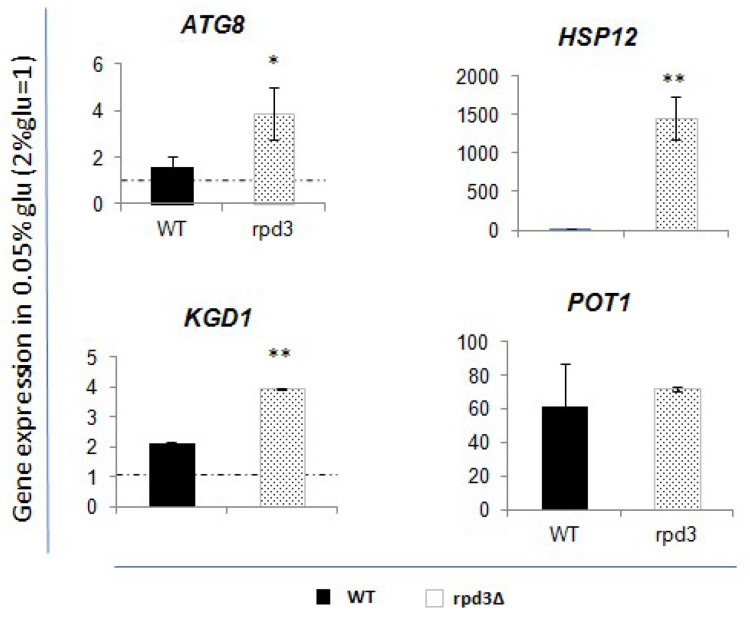
Expression rate of the analysed genes after glucose deprivation (0.05% glucose) in rpd3∆ strain, compared to WT values. Fold increase is calculated respect to ACT1 expression. Dashed lines represent expression level before glucose deprivation (2% glucose). Black bar is relative to the WT strain values; dotted grey bar represents the rpd3∆ values. The values and statistics were calculated from three biological replicates. Significance was assessed using Student’s *t*-test. * *p* ≤ 0.05, ** *p* ≤ 0.01.

In Figure 6, we show the expression levels of *ATG8*, *HSP12*, *KGD1*, and *POT1*.

The data indicate that, after glucose deprivation, the *rpd3* deletion induces a more pronounced upregulation of *ATG8*, *HSP12*, and *KGD1* compared to what is observed in WT cells. *POT1* does not appear to be significantly affected by the absence of Rpd3p.

### 3.7. Rpd3p Recruitment at Promoters During Low Glucose-Induced Stress Depends on Top1p

In line with the idea that Rpd3p could be responsible for repression of genes during the WT cell stress response, we investigated the presence of Rpd3p at promoter regions of *ATG8*, *HSP12*, *KGD1* genes, which showed an increased upregulation in the *RPD3*-deleted context. *POT1* was also investigated as control, given that *rpd3* deletion seems to have no interference with its expression level after glucose decrease. We aimed to determine whether the amount of Rpd3p varies when genes are stimulated by glucose deprivation.

As performed for all analyses described in this work, cells were grown in a medium containing standard glucose concentration (2%) and then compared with those shifted to a medium with low glucose (0.05%) for three hours. Cells were collected and subjected to ChIP analysis according to the protocols described in the Materials and Methods section. The results obtained for the “low glucose” samples were compared to those obtained for the corresponding “standard glucose” samples. ∆Ct values were calculated between the immunoprecipitated sample and the INPUT sample. Then, fold increases of the “low glucose” samples were normalized to the “standard glucose” samples. The *INO1* gene was used as a reporter for Rpd3 immunoprecipitation, as described in literature [16].

**Figure 7:**
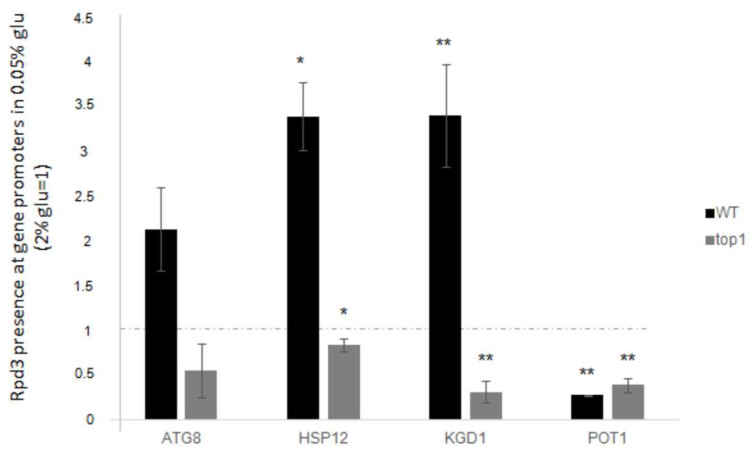
Rpd3 enrichment at promoters of all the analysed genes after glucose deprivation (0.05% glucose). Fold increases are calculated respect to ARS504 locus and to relative INPUT. Dashed line represents Rpd3 presence before the glucose deprivation (2% glucose). Black bars: WT strain; grey bars: top1∆ strain. The values and statistics were calculated from three biological replicates. Significance was assessed using Student’s *t*-test. * *p* ≤ 0.05, ** *p* ≤ 0.01.

Figure 7 (black histograms) shows levels of the Rpd3p recruitment at genes promoters in WT cells after glucose deprivation (relative to standard glucose data).

On the other hand, as previously described, it is well known that Top1p can act as a scaffold protein to attract regulatory factors [4,5,8,24,25,26]. Considering these two ideas, we aimed to determine whether Top1p could coordinate gene expression regulatory activities by guiding Rpd3p.

To investigate this, we quantified the presence of Rpd3p on promoters after low glucose stress in *top1∆* cells and compared the results to those obtained with the WT strain. The results shown in Figure 7, grey histograms, indicate that at the *ATG8*, *HSP12*, and *KGD1* promoters the presence of Rpd3 protein drastically decreases in *top1∆* cells compared to WT. Conversely, as expected, when Rpd3p occupancy at *POT1* promoter was analysed, we observed the same behaviour as in the WT strain.

Data describe that the presence of Rpd3p on *ATG8*, *HSP12*, and *KGD1* promoters increases by two to four-fold when low glucose stress is applied, compared to standard glucose. Conversely, Rpd3p is not observed to bind the *POT1* promoter region, as expected from the expression result shown in Figure 6 concerning this gene.

## 4. Discussion

The stress genes we studied in this work increase their transcription after reduction of glucose in the culture medium. When we studied this response in the *top1Δ* strain, a further increased transcription is observed compared to WT, in the same low glucose condition (Figure 1). This phenomenon suggests a repressive- like mechanism that DNA topoisomerase 1 could exert on the expression of the studied stress genes. DNA topoisomerase 1 can exert a repressive role on transcription, and it has been observed both during the stationary phase [6] and on rDNA silencing [7,8].

Associated with the observed transcriptional increase after decrease of glucose in the medium, we have classically observed H3 hyperacetylation at the promoters chromatin, with further increase of this modification when the process is studied in the top1 strain (Figure 2).

Since 1993 [4,5] and then with subsequent works [8] it has been demonstrated that DNA topoisomerase 1 can act in the control of transcription regardless of its catalytic activity. This way of altering transcriptional control is mostly based on the function as a scaffold protein. Top1 recruits transcriptional regulators appropriately to its positive or negative regulatory role. Even in the regulation of the stress genes analyzed in this work, it seems that the catalytic function is neither required for the repressive action on transcription (Figure 3), nor for the associated H3 acetylation (Figure 4). This suggests that Top1p might also act as scaffold protein in the regulation of these genes. However, the reality is more complex. In fact, when we measured the presence of Top1p at the promoters of the stress genes analyzed, we did not observe significant differences associated with the transcriptional state (Figure 5). This suggests that the DNA topoisomerase 1 is stably bound to these regions regardless of the transcriptional state. However, it must be considered that the technique we used could hardly distinguish the boundary between promoter and coding region where it has been demonstrated that Top1 interacts regardless of transcription [30]. It is also reasonable to think that the protein could reside permanently at the promoter areas due to its topological role. A similar condition has been well demonstrated at 37S rDNA promoter, where DNA topoisomerase 1 stably resides [15]. Another hypothesis that could be considered is the possible presence of sequences and/or specifical 3D structure formation that particularly attract it.

Given this relative relevance of Top1 on promoters, we wondered whether one of its interactors could be recruited at the genes, and be responsible for their activation/repression. The interactomics data show that Top1 is involved in an high number of interactions (592 genetic interactions and 74 physical interactions according to the Saccharomyces Genome Database). Among the candidates suitable for the role of a possible transcriptional regulator we considered Rpd3 histone deacetylase. Both transcriptional repression and activation processes are attributed to this histone deacetylase, depending on its recruitment within the “Large” (negative transcription regulator) or “Small” (positive transcription regulator) complexes. Furthermore, the Pho23 protein, which contributes to the incorporation of Rpd3p within the Large complex [28], is found to be also included among the interactors of Top1p [29].

We then proceeded with the measurement of the transcription levels of the genes, after lowering glucose in the medium, in a mutant strain due to deletion of the *RPD3* gene, coding for the histone deacetylase Rpd3. The data collected allowed us to observe three genes in particular: *ATG8*, *HSP12*, *KGD1*, for which the transcriptional increase due to low glucose is exacerbated by the absence of *RPD3*, making us hypothesize that Rpd3p may act as a negative regulator of expression (Figure 6).

In support of the role of Rpd3p as a possible negative regulator of the expression of *ATG8*, *HSP12* and *KGD1*, we detected an increase in the protein on the promoters when the genes are activated and furthermore the recruitment of Rpd3p seems to be directly related to the physical presence of Top1p, in fact the lack of DNA topoisomerase 1 (*top1Δ* strain) in addition to increasing transcriptional induction, causes the lack of recruitment of Rpd3p to the observed promoters (Figure 7).

Taking together all these observations, we formulated this model: lowering glucose in the medium is a transcription activation signal that must be both timed and strictly correlated to the stress signal that comes from the environment (low glucose). Therefore, through Top1p, Rpd3p is recruited as a negative regulator on promoters, so that gene activation shall not exceed the limits necessary for cell response to environmental stress and can quickly return to constitutive expression levels.

Conversely, *POT1* induction after glucose deprivation is not dependent on the “Top1p-Rpd3p pattern”. In our observations its gene activation is not affected by deletion of *RPD3*, not significantly affected by *TOP1* deletion, and moreover Rpd3p seems to be not recruited to the promoter region after low glucose induced transcription activation.

Thus, what attracts Top1p to the promoters of stress genes? Into the *HSP12* promoter (437/-430 from the ATG) we found the STRE sequences (TAAGGGG, core: AGGGG [31]). This sequence can potentially undergo the 4G transition, which is an alternative DNA conformation known to be more attractive for Top1p [32].

Observing *ATG8* and *KGD1* promoters, the canonical STRE sequence is absent, but the detection of many repeats of Gs have led us to suppose that the region may be structurally inclined to form G-quadruplex. In order to validate this hypothesis, we submitted the promoter sequences of interest to the G4 hounter- DNA analyzer web application, a software which allow to predict the propensity of certain sequences to form G- quadruplex [33].

Thanks to this prediction analysis we found that both on the *HSP12* and *KGD1* promoters there are regions with a high probability of forming G- quadruplex structures. Therefore in our model Top1p is linked to the promoters of stress genes thanks to the presence of attractive three-dimensional structures.

These observations regarding the ability of topoisomerase 1 to recruit transcriptional regulators, including epigenetic ones, reinforce the hypothesis that Top1p is a fundamental component of the transcription process, capable of operating in different ways: relieving torsional stress, recruiting regulators, and focusing on different regions depending on the potential alternative structures that may form on the DNA. This perspective further broadens the scope of action of this essential and versatile enzyme.

## 5. Conclusions

When the stress genes *ATG8*, *HSP12*, *KGD1*, and *POT1* are induced to transcribe by lowering glucose levels, the transcriptional response is intense but controlled. In fact, if the same induction is performed in the *top1∆* strain, the response is much higher than in the WT. This transcriptional control effect promoted by Top1p does not require its catalytic activity but presumably involves the recruitment of the *RPD3* factor. Indeed, in the *rpd3∆* mutant, the transcription is similar to that of the *top1∆* strain. In the *top1∆* strain, Rpd3p does not associate with the promoters. *POT1* does not follow this pattern. From these data, we conclude that Top1p, regardless of its catalytic activity, is attracted to the promoters of these genes by recruiting Rpd3p and maintaining transcription at a controlled rather than an extreme level, as occurs in the absence of *top1∆* or *rpd3∆*. This work further emphasizes the role of Top1p that goes beyond its catalytic activity.

## Data Availability

The data presented in this study are available in the manuscript.
